# Job satisfaction behind motivation: An empirical study in public health workers

**DOI:** 10.1016/j.heliyon.2021.e06857

**Published:** 2021-04-21

**Authors:** Fotis Kitsios, Maria Kamariotou

**Affiliations:** Department of Applied Informatics, University of Macedonia, Thessaloniki, Greece

**Keywords:** Motivation, Job satisfaction, Performance, Health personnel, Healthcare

## Abstract

The health sector is characterized as labor-intensive, which means that the effectiveness of an organization that operates within its context is inextricably linked to the level of employee performance. Therefore, an essential condition, in order to achieve higher standards, in terms of the effectiveness of the health units, as well as set the foundations of a solid health system, is to take maximum advantage of the full potential of human resources. This goal can only be accomplished by providing the appropriate incentives, which will naturally cause the adoption of the desired attitude and behavior. In the case of Greece, there is not enough research relative to the needs of health workers and, consequently, the incentives that can motivate them. This article aims to investigate the dynamics that may be behind health workers at a public hospital in Northern Greece. Data were collected from 74 employees in the hospital and were analyzed using ANOVA analysis. The results show that key motivators for the employees can be considered the relationships with their colleagues and the level of achievement, while the level of rewards and job characteristics play a secondary role. These results make it clear that, in order for the hospital's management to be able to improve the level of employee performance, it should ensure the establishment of a strong climate among employees, and also acknowledge the efforts made by them.

## 1 Introduction

The field of health is particularly complex as it focuses on the provision of health services, which are mainly produced by the human resources that staff the health units. The quality of the services under study is largely determined by the behavior of health workers as a result of their efforts (Musinguzi et al., 2018). For this reason, the administrations of the health units must give great importance to the utilization of the human factor, to increase their effectiveness and, consequently, the effectiveness of the health units. In order for healthcare workers to be efficient and provide patients with superior quality services, the followings are necessary: health workers must have clear expectations of the subject of their work and their work environment, they must have the appropriate knowledge and skills needed for their work, they must have access to necessary equipment, receive feedback on their performance and have a supervisor who motivates them. More simply, healthcare workers need incentives to motivate them in order for them to satisfy patients and increase their effectiveness (Hotchkiss et al., 2015; Huber and Schubert, 2019; Schopman et al., 2017).

Even in those cases where health workers do not have the proper means and equipment, incentives given to them seem to help them overcome these problems and attend to patients to the fullest. Popa and Popescu (2013) and Rossidis et al. (2016) argue that meeting the needs of employees through the provision of incentives is a powerful tool that administrations can use to motivate them and increase their productivity. This is an element that all health organizations should take advantage of, as this can help them to deal with and overcome serious problems that limit their effectiveness (Borst et al., 2020; Valdez and Nichols, 2013). In this context, it is important to state that motivating healthcare workers is an urgent need as it increases their performance and consequently increases the efficiency of the services provided as well as patients’ satisfaction (Hotchkiss et al., 2015; Rubel et al., 2020).

The present research aims to examine the factors that may motivate employees at a public hospital in Northern Greece. The need to provide incentives for healthcare workers in order to increase their performance is urgent, as the health systems of several countries have been severely affected bythe global financial crisis, which must be overcome. Reducing the number of health care workers coupled with shortages of material and technological equipment limits the efficiency and effectiveness of health care units, making it necessary for their administrations to look for a way out. The management of health units, in order to achieve effective management of available resources and produce higher quality health services, must make the best use of the human factor.

Essentially, the administrations of the Greek health units must design realistic incentive programs for their employees so that they can increase their efficiency. The provision of incentives to health workers is necessary for them to meet their needs. This means that healthcare management needs to know what motivates its staff to meet their needs and also needs to know whether their needs differ based on their personality type and profession. The results of this work are expected to be particularly useful and collaborate with other research work done during the last years of the economic crisis on the needs of health employees and knowing the incentives that can motivate them.

The research questions posed for the present research work to answer are:

1. What are the internal factors that motivate health professionals and the administrative staff of the hospital?
2. What are the external factors that motivate health professionals and the administrative staff of the hospital?

This paper is divided into sections: section 1 introduction of the subject, section 2 presents the theoretical background about the motivation of healthcare workers. Section 3 is the methodology, Section 4 presentation of research findings and Section 5 comments on the research results and the conclusions of the study.

## 2 Theory

### 2.1 Hospital management

The management of hospitals is very important for their proper functioning. It is also worth noting that hospital management differs from that of other sectors. Additionally, there are important differences between the management of private and public hospital management. The main goal of the management of private health units is to achieve profit and reduce costs and required resources. On the other hand, the main purpose of public hospitals is to promote and provide health services to all citizens without discrimination and exclusion criteria. There is also a great deal of effort in public hospitals to increase the quality of services provided, which often leads to a waste of available resources, creating problems for public health units (Masango-Muzindutsi et al., 2018; Musinguzi et al., 2018).

In public hospitals, due to the difficult economic conditions prevalent in this country, there are problems in the quality of services provided. Great efforts are being made to reduce the cost of health services without quality being compromised. The measures taken by governments include attempts to increase the efficiency and effectiveness of public organizations, and rational use of the limited resources available. Thus, it is necessary to have the appropriate management in the health sector, which will be able to use resources rationally and in particular to coordinate the human resources in the most effective way to increase the quality of services provided in public health units (Aysen and Parumasur, 2011; Liu et al., 2015). Also, in modernizing the administration of the public health units, efforts should be made to eliminate the pathogenesis of the Greek health system and reduce its weaknesses that have existed for years. It is worth noting that the management of public hospitals should be free from influences such as government policy so that it can carry out its work in the best possible way and be guided by the social interest and service of the citizens.

It has been found that leadership is very important in creating the necessary conditions within an organization to successfully achieve its goals. In essence, leadership is influenced by the reactions and behaviors of employees in relation to organizational change, the performance of the business as a whole, job satisfaction of staff and the culture that prevails within an organization. A leader, based on his values and characteristics, can positively or negatively affect employees and their behavior. Every leader, based on his or her skills and abilities, makes appropriate decisions and influences the way employees work and is also responsible for the elimination and proper management of conflicts and issues that may arise in the business (Belrhiti et al., 2020; Curtis & O'Connell, 2011).

If a leader is effective, he or she can create an attractive vision for employees and inspire them to pursue and work toward the success of their goals. Also, a leader is effective when he can motivate his subordinates and make them committed to achieving their goals. Other actions of an effective and successful leader include the creation of performance and reward criteria, and the creation of effective teams and modes of communication between employees (Musinguzi et al., 2018; Rubel et al., 2020).

Several factors have been reported to increase the job satisfaction of health care workers. One of the factors that contribute to the increase in staff satisfaction is their salary and wages. In fact, due to the pathogenesis of the Greek health system as well as their economic crisis and cuts in health care expenditures, there have been reductions in staff's salaries, invalid payments of medical and nursing staff and non-payment of various allowances in this profession. These factors lead to a reduction in healthcare staff's job satisfaction (Halldorsdottir et al., 2018; Kontodimopoulos et al., 2009).

To improve the job satisfaction of medical and nursing staff as well as productivity, there could be some level of reward. Thus, each health care worker could be more productive if there is a corresponding reward that would lead to an increase in his/her job satisfaction. This can make the entire performance of the whole health unit to increase, which in turn would increase the quality of services provided (Borst et al., 2020; Kontodimopoulos et al., 2009; Rubel et al., 2020).

Equally important is the fact that there is an increased sense of job satisfaction in workers at health organizations and a sense of justice within the unit. Meritocracy, objective judgment on promotions and salary increases, rational division of duties and work within the unit, fair distribution of shifts and impartial attitude of the administration are significant factors that can influence job satisfaction of nursing and medical staff (Kontodimopoulos et al., 2009; Schopman et al., 2017). These factors can contribute to the increase in employees’ satisfaction, and as will be analyzed in the next section, they can also be incentives for employees in health units.

Other important factors that can lead to increased job satisfaction of healthcare workers are factors related to working conditions and the work environment. In particular, these factors include the existence of comfortable and functional workplaces and staff rest, the existence of appropriate equipment and consumables and the relationships among employees and those between employees with management. The factors that boost job satisfaction of medical and nursing staff include good communication that should be reciprocal, teamwork, and cooperation as well as the existence of good relations and respect within the health unit (Kontodimopoulos et al., 2009; Schopman et al., 2017).

Another factor that increases the job satisfaction of healthcare employees is the acknowledgment of their work and appreciation they receive from their colleagues, management and patients. When the work of employees is acknowledged, they have increased levels of job satisfaction (Borst et al., 2020; [Bibr bib22][Bibr bib22]). Aside the acknowledgment of their work, employees are highly satisfied with their job when there are opportunities for development and growth within the health unit (Lambrou et al., 2010). There is a need for these conditions to be present in the health units to increase the job satisfaction of their staff.

Other important factors that increase employees’ job satisfaction include the assignment of important responsibilities to them and allowing them to participate in the decision-making process (Lambrou et al., 2010; Schopman et al., 2017). This will make them to feel that it is important to carry out important decisions and actions within the unit, thereby increasing their job satisfaction. From the above, the management and the leadership in each health unit should take into account the factors that can increase the job satisfaction of their employees. In this way, they will be able to increase their efficiency and productivity and make the health unit work much more efficient and successful, increasing the satisfaction of health service users.

### 2.2 Encouraging healthcare workers

In recent years, the interest in motivating health workers has been particularly strong as it seems to be crucial to the quality and effectiveness of the services provided. Rosak-Szyrocka (2015) argues that motivating health workers is a necessary and important process as it ensures their commitment both to their organization and the work they produce. As a result, the quality of the work of health and administrative staff is increasing. Therefore, it is expected that patients will receive not only satisfaction from the services they receive but also from the relationships they develop with doctors, nurses and the administrative staff of the health units. The effect of health workers' motivation on the quality of health care provided to patients is also confirmed by Alhassan et al. (2013) and Babic et al. (2014). In particular, Alhassan et al. (2013), through their research, showed that health workers who are not motivated by the administrations of the health units in which they are employed are very likely to provide unsafe health services.

Tsounis et al. (2013), through a literature review, concluded that healthcare workers may not perceive incentives the way employees in other sectors do. They also argue that the individual categories of healthcare workers can be motivated in different ways and degrees due to their different needs and requirements. Typically, financial rewards are not an incentive for doctors to increase their performance. This is contrary to what happens in many different industries and also with other categories of healthcare workers. Instead, doctors seem to be motivated when they meet their goals and are acknowledged by both hospital management and their colleagues.

Identifying the factors that motivate healthcare workers has also been a significant subject of research by Babic et al. (2014). They tried to identify whether there are differences between the incentives that motivate employees in the private health sector and those in the public health sector. The results of the survey showed that not all employees are motivated by material rewards as they are very low and employees in the private sector are positively motivated by the existence of security conditions. Also, in both private and public sectors, healthcare workers seem to be positively motivated by peer relationships and the support that develops between them. In contrast, employees in both private and public sectors do not seem to be satisfied with the way their superiors assess them, which does not help to motivate them. The results are moving in the same direction regarding the development incentives provided to both private and public health sectors, which are very limited and cannot motivate healthcare workers to increase their performance (Mariappan, 2013).

Bhatnagar et al. (2017) argue that various motivations can lead to the promotion of healthcare workers, which can be either internal or external. Characteristically, they argue that their faith, values and self-efficacy are internal factors that influence or motivate them; economic gains and working conditions are external motivations that can affect their performance. Lambrou et al. (2010), in their study, tried to investigate the factors that can motivate healthcare workers to adopt the desired behavior. The results of the research showed that salary, the relations between the colleagues as well as the nature of the work, are the determining factors of their motivation.

Hotchkiss et al. (2015) also researched on the incentives that health unit administrations can provide to employees to increase their effectiveness. They found that being motivated by both internal and external factors influences the effectiveness of workers. More specifically, employees' self-efficacy and self-esteem are two of the main internal motivations that motivate healthcare workers. It also emerged that in Ethiopia, healthcare workers’ satisfaction with their financial earnings, working conditions and relationships with their colleagues are the main external motivations that can motivate them.

Dagne et al. (2015) conducted another study in Ethiopia that tried to explain the factors that drive healthcare workers. The results of this research showed that healthcare workers are primarily motivated by their superiors and the relationship they create with them, by financial rewards, the nature of the work and the tasks they undertake and the location of the hospital where they work.

In addition, Shah et al. (2016), in their research, concluded that the main factors that can motivate employees in the field of health are the safety of the work environment, recognition and rewarding of their work, provision of incentives and the possibility of their development within work.

Janus et al. (2008) identified various incentives that can be offered by healthcare unit management so that healthcare workers can be motivated and increase their effectiveness. Such incentives are participation in the decision-making process, participation in educational programs, the existence of security conditions in the work environment, good relations between colleagues as well as fair treatment given by their superiors. However, the most important incentives seem to be the financial rewards provided to healthcare workers by the management of health facilities (Alotaibi et al., 2016; Halldorsdottir et al., 2018).

In a bid to examine the importance of motivating health workers in Greece, Gaki et al. (2013) investigated the factors that can motivate the nurses of Greek hospitals. The study involved 200 nurses who argued that they cannot be motivated only by material rewards (financial rewards) but also by factors that contribute to their personal and professional development. They also argued that their motivating factor is the job satisfaction they receive both from the exercise of their duties and from the conditions prevailing in their work environment. Holmberg et al. (2016) found the same results in a survey conducting in a Swedish clinical nursing staff.

### 2.3 Differentiation of incentives according to the personal and professional characteristics of health workers

A particularly interesting study is that of Rosak-Szyrocka (2015), which makes it clear that gender is a significant factor in differentiating the health needs of workers and therefore the motivations of those who positively influence their behavior. More specifically, he argues that perceptions and social stereotypes affect their needs and thus seek different motivations to be able to be motivated. Lambrou et al. (2010) to this; it makes it clear that female health workers are more easily motivated by salary compared to men. Goncharuk (2018) found that the most significant motivator for women is salary but for men is the characteristics of their job.

Like the above researchers, Shah et al. (2016) found that there is a difference between the factors that motivate women and men who work in the health sector, making it clear that motivating women is a more difficult process. Like gender, the age of healthcare workers seems to be a determining factor in differentiating the motivations that can lead to the promotion of healthcare workers (Gaki et al., 2013; Kantek et al., 2015; Park and Lee, 2020). Contrary to the above research, the research of Babic et al. (2014) did not identify the existence of different needs between the sexes. Babic et al. (2014) concluded that demographic characteristics do not affect employees' views on those factors that may affect their performance and actually motivate them. Similarly Dagne et al. (2015) found no significant statistical relationship between the demographic characteristics of the sample and the factors that motivate them.

The study led by Toode et al. (2015) provided additional support for the assertion that demographic factors for example age, working experience, position and educational training influence nurses’ motivation. They found that older and more tenured nurses were more probable motivated by external reasons. For managers, this is a particularly significant outcome to underline and brings up the issue of how to help and keep up intrinsic motivation when staff get older and have worked longer in medical care. Another important outcome is that workers who have had received limited training during the most recent years were less motivated than colleagues who were accomplished. Concerning family life, having children and whether the staff members lived alone or with a partner had no relation to their work motivation.

It is also important to note that the position of employees in the health unit affects their needs and requires the formation of different policies on the part of management to motivate them. Tsounis et al. (2013) argue that physicians are motivated by different factors and motivations compared to nurses and administrative staff. Similarly, Lambrou et al. (2010) showed that nurses are more interested in motivations that relate to material rewards than physicians who are interested in other motivations as stated earlier on. The highest ranked factor for nurses’ motivation is “achievements” according to Grammatikopoulos et al. (2013) which conducted in Greece.

The following hypotheses have been identified on the basis of existing studies:

H1 Job related factors positively affect healthcare professionals' satisfaction.

H2 Factors related to salary positively affect healthcare professionals' satisfaction.

H3 Factors related to relationships with colleagues positively affect healthcare professionals' satisfaction.

H4 Factors related to work achievements positively affect healthcare professionals' satisfaction.

## 3 Materials and methods

The research is carried out on the employees of a public hospital in Northern Greece and specifically, on the administrative employees, nurses and doctors. This means that the research population is the staff of the hospital under study, which amounts to 947 people. The study involved 74 hospital staff as the number of correctly completed questionnaires collected. It is important to note that 150 questionnaires were distributed, of which 11 were incorrectly completed and 65 were not completed at all, indicating a relatively high reluctance to participate in the survey.

The questionnaire is based on Paleologou et al. (2006) and it includes 4 categories of factors in order to examine the level of workers’ motivation in healthcare. These factors are related to job, salary, relationships with colleagues and work achievements. A 5point Likert ordinal satisfaction scale was used. Appendix displays the items as they appeared in the survey. Data were analyzed using ANOVA.

This paper does not involve chemicals; procedures or equipment that have any unusual hazards inherent in their use. Furthermore, it does not include experiments that may raise biosecurity concerns or human subjects or a clinical trial. Finally, the paper does not involve experimentation on animals.

## 4 Results

Majority of the 74 employees were women (n = 60, 81.08%) while a smaller percentage were men (n = 14, 18.92%). 37.84% (n = 28) of the participants were aged 36–45 years, 36.48% (n = 27) of the participants were aged 46–55 years, 17.57% (n = 13) of the participants were aged 26–35 years and 8.11% (n = 6) of the participants were aged 18–25 years. 43.24% (n = 32) of the participants were graduates form Technological Institutes, 29.73% (n = 22) of the participants were high school graduates, 8.11% (n = 6) were postgraduate degree holders, 4.05% (n = 3) were graduates from Universities, 2.7% (n = 2) were school graduates and only 12.16% (n = 9) stated another level of education. The vast majority were health professionals (n = 73, 98.65%) and only 1 person (1.35%) was an executive staff (Table 1).

**Table 1:** Descriptive statistics of the sample.

Variable	Sample	Percentage (%)
Gender:		
Men	14	18.92%
Women	60	81.08%
Age:		
18–25	6	8.11%
26–35	13	17.57%
36–45	28	37.84%
46–55	27	36.48%
>=56	0	0.00%
Education:		
School graduates	2	2.70%
High school graduates	22	29.73%
Graduates from Universities	3	4.05%
Graduates form Technological Institutes	32	43.24%
Postgraduate degree holders	6	8.11%
Other	9	12.16%
Marital status:		
Unmarried	14	18.92%
Married	43	58.11%
Divorced	16	21.62%
Other	1	1.35%
Position:		
Health professionals	73	98.65%
Executive staff	1	1.35%

Internal consistency was calculated via Cronbach's alpha with the minimum requirement of 0.70 (Newkirk et al., 2003). The Cronbach's alpha coefficient values for the variables are calculated in Table 2.

**Table 2:** Reliability analysis of the questionnaire items.

Variables	No. of items	Cronbach a
Factors related to employment	11	0.848
Factors related to salary	4	0.670
Factors related to relationships with colleagues	8	0.861
Factors related to achievement	6	0.859

The outcomes of Pearson's correlation analysis done to ascertain the level and type (direct or inverse) of relationship amongst the variables are presented in Table 3.

**Table 3:** Correlation matrix.

	Job factors	Salary	Relationship with colleagues	Work achievement	Satisfaction
Job factors	1	0.592	0.626	0.546	0.183
Salary	0.592	1	0.633	0.552	0.183
Relationship with colleagues	0.626	0.633	1	0.792	0.258
Work achievement	0.546	0.552	0.792	1	0.418
Satisfaction	0.183	0.183	0.258	0.418	1

**Figure 1:**
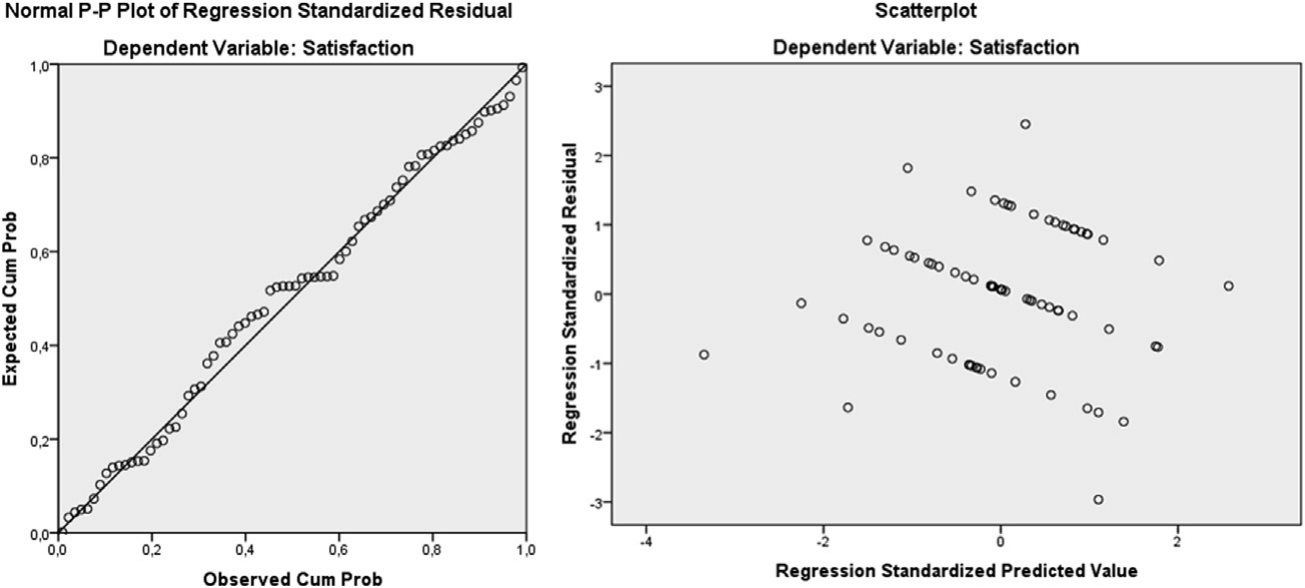
Standard P–P plot of the regression standardized residual and a residual scatter plot.

From the normal P–P and scatter plots (Figure 1), the data are usually distributed (all residuals cluster around the ‘line’) and conform with the assumptions of homogeneity of variance (homo-scedasticity) and linearity. The residual errors are evenly spread and not linked to the predicted value, thereby suggesting that the correlation is linear, and the variance of y is the same among all values of x, which supports the homoscedasticity assumption (Kachigan, 1991). Z-score was used to evaluate the univariate outliers and all values were within the acceptable range. Mahalanobis and Cook's distances were used to evaluate the multivariate outliers. No influential outliers were identified. Variance inflation factors (VIFs) was used to evaluate Multicollinearity.

Table 4 presents the regression coefficients and the results of hypothesis testing. The path coefficient between job factors and satisfaction was negative and not statistically significant (β = -0.008, p > 0.05). Therefore, H1 was not supported. There was a negative and not statistically relationship between the factors that are related to salary and satisfaction (β = -0.012, p > 0.05). Therefore, H2 was not supported. There was a negative and not statistically significant relationship between the factors that are related to relationship with colleagues and satisfaction (β = -0.186, p > 0.05). Thus, H3 was not supported. There was a positive and statistically significant relationship between the factors that are related to work achievements and satisfaction (β = 0.577, p < 0.05). Thus, H4 was supported.

**Table 4:** Regression analysis between independent variables and dependent variable.

Model	β	t-Value	VIF
Job factors	-0.008	-0.057	1.850
Salary	-0.012	-0.083	1.876
Relationship with colleagues	-0.186	-0.933	3.377
Work achievement	0.577	3.222	2.729

∗p < 0.05, ∗∗p < 0.01, ∗∗∗p < 0.001.

## 5 Discussion

The findings show that the majority of the sample is moderately satisfied with the exercise of power within the hospital as well as moderately satisfied with the clarity of their work objectives. However, it should be noted that there is a significant percentage of respondents who are not at all satisfied with the clarity of their work goals. This raises significant concerns as these employees do not have a goal to guide their actions and behavior. Also, a large part of the sample states moderate to good satisfaction with the clarity of their duties, but again several employees do not seem to understand what their obligations are.

The majority of the survey participants are moderately satisfied with the safety of their work environment. A large part of the participants believes that their potential cannot be maximized as they are not provided with the appropriate opportunities. Regarding the satisfaction they receive from the conditions prevailing in the work environment of the hospital, majority of the sample statement is very satisfied, which is very encouraging as it seems that there is a positive atmosphere; however, all the employees are not very satisfied with the resources of the hospital in order to be able to perform their work.

Regarding the possibilities of personal and professional development through the educational programs provided to the employees of the hospital, majority of the sample and specifically almost half of the participants expressed moderate satisfaction. This makes it clear that there are significant opportunities for improvement as job development and training opportunities must be provided to employees in order to improve their skills and abilities. Also, it seems that most of the participants are moderately satisfied with the ability to exercise control over the decisions made and with the ability to participate in the decision-making process. In this regard, with the satisfaction that employees receive from their job position, several employees show little satisfaction, several employees who show great satisfaction and finally many who show great satisfaction.

Of particular interest are the views of employees at the Hospital regarding the satisfaction they receive with the achievements they make. Typically, majority of the sample claim to be moderate to very satisfied with the importance of their work as well as the levels of respect that exist between hospital staff. A large part of the participants claims that they receive moderate to significant satisfaction from the recognition of the efforts they make and the achievements they make. However, majority of the participants believe that there are no suitable opportunities for personal growth and development in the hospital. Of particular interest is the finding of satisfaction with the incentive effort adopted by the administration as the majority of the sample is not at all satisfied with this process or is at least satisfied with it. This is a finding that makes it necessary to change the attitude and behavior of management regarding the way employees are treated as they need to adopt more effective techniques that will contribute to their effective motivation.

The satisfaction of the participants in this study regarding the external incentives provided to them, in particular, the remuneration they receive and their relations with their colleagues was investigated. The results showed that majority of the sample are not at all even slightly satisfied with the financial earnings they receive and moderately satisfied with the benefits of pension and insurance coverage. However, they are moderately satisfied with their work environment and not at all satisfied with the policies for dealing with absenteeism.

In contrast to the above incentive categories, where there seems to be overall moderate satisfaction, in the case of relationships with colleagues, the results differ significantly: majority of the sample are moderate to very satisfied with the interpersonal relationships developed in their work, by the existence of a spirit of teamwork and cooperation, stimulation of pride and respect, appreciation of the role of employees, the support they receive from supervisory staff and supervisors, the fair treatment they receive and the ability they have to express themselves creatively. Finally, most of the hospital staff members are very satisfied with their relationship with their colleagues (Ayalew et al., 2019; Belrhiti et al., 2020; Chmielewska et al., 2020; Huber and Schubert, 2019).

Regarding the results of those factors that drive hospital staff, it became clear that the main factor is the relationship with their colleagues and the achievements that follow. Both the salary and the characteristics of the job position are not the main motivating factors for the employees at the hospital. Similarly, Hotchkiss et al. (2015) and Lambrou et al. (2010) found that relationships between colleagues are a major motivator for health workers. The finding that differs from the aforementioned surveys concerns their remuneration which seems to be a key motivating factor, which is not confirmed by this research.

The increase in hospitalization and the prices of medicines and the reductions in the state health budget have led to significant problems in the health sector. Citizens can no longer afford private insurance thereby increase the workload in public hospitals. Unemployment has also risen. There is an increased social exclusion of the unemployed or those who do not have a job, leading to increased psychological disorders of individuals. Unemployment has even led to an increase in illness and psychological problems, as well as the number of disability and suicide cases. Thus, the public sector cannot effectively meet the health needs of citizens in a quality manner (Potipiroon and Faerman, 2020). Respectively, health professionals cannot meet the particularly high needs of citizens and therefore their job satisfaction is reduced; there is also a strong brain drain phenomenon, during which many health professionals go abroad.

It is also important to mention that the process of financing the Greek public sector cannot meet the ever-increasing needs that exist, and therefore the proper functioning of public hospitals is affected. In fact, due to the shortages of material, consumables and equipment, the work of health professionals has become much more difficult in recent years (Stuckler et al., 2009). Also, the economic crisis has led to a reduction in the income of citizens which has in turn led to a restriction on the use of health services by citizens, increasing the workload in public Greek hospitals. Thus, the pathogenesis of the Greek health system combined with the reduction of state health budgets has led to even more problems for health professionals and a reduction in job satisfaction. There is a need to provide the right incentives to increase the quality of health services.

## 6 Conclusion

The aim of this paper was to investigate internal and external factors that may motivate employees at a public hospital in Northern Greece. The results of this paper point out that without a strong motivational plan, the inner incentive of hospital nurses to work can become an extrinsic motivation over time. Managers should recognize and note when their nursing staff, who have been dedicated to self-focused for many years, require additional help or improvement in their position and be sensitive to giving them the extra assistance they may need to stay effective and independent.

The results of this paper can be used by the hospital to formulate more effective strategies, as they show that health workers are not fully satisfied with the prevailing conditions and that improvements need to be made to produce better quality health services. As it turned out, healthcare workers are not particularly happy with the level of knowledge of the strategic goals of healthcare units and their tasks, as well as the opportunities given to them to participate in decision-making processes and to develop personally and professionally. This means that the administrations will have to adopt a different leadership that will emphasize the needs of the employees and allows them to develop a more active role within the health unit. They should also encourage the creation of educational programs and create conditions for development so that hospital employees know that their work encourages their development and gives them opportunities for advancement.

It also became clear that hospital staff is more motivated by the good relations that exist between colleagues. This means that the management of the hospital should ensure in every way that there is a positive atmosphere of cooperation between its staff members and that there is a strong element of mutual support. It must look for all ways to create harmonious cooperation among members of the organization as well as strong interpersonal relationships and ties. Otherwise, employees cannot be productive and will not be able to provide quality health services, which are necessary for the citizens of the country, especially in such a difficult time.

The results of this work are of great interest, but cannot be generalized to the entire population of hospital staff as the sample involved in the study came from only one hospital in the country. It should also be noted that the results of the survey were obtained from a small sample of participants. These data make it clear that it is necessary to conduct a future survey of a larger population of health workers, from different parts of the country, to determine the situation in the whole country and to investigate the factors that motivate the staff to acquire the appropriate attitude and behavior needed for work. Through such future research, all hospital administrations in the country will know the needs of their employees as well as what motivates them. Besides, the paper gives some useful materialness to inspect medical attendants' work inspiration qualities. The questionnaire can be used to design a number of effective interview methods and spotlight on explicit individual mediations that can build the degree of motivation in a specific position or work unit.

## Declarations

### Author Contribution statement

Maria Kamariotou: Analyzed and interpreted the data; Contributed reagents, materials, analysis tools or data; Wrote the paper.

Fotis Kitsios: Conceived and designed the experiments; Performed the experiments; Contributed reagents, materials, analysis tools or data; Wrote the paper.

### Funding statement

This research did not receive any specific grant from funding agencies in the public, commercial, or not-for-profit sectors.

### Data availability statement

The data that has been used is confidential.

### Declaration of interests statement

The authors declare no conflict of interest.

### Additional information

No additional information is available for this paper.

## Figures and Tables

**Table:** 

Job factors	Very dissatisfied (1)-Very satisfied (5)
How satisfied are you with exercising power?	
How satisfied are you with the clarity of the goals in your work?	
How satisfied are you with the clarity of the tasks at work?	
How satisfied are you with your workplace safety?	
How satisfied are you with your skills?	
How satisfied are you with the adequacy of your work resources?	
How satisfied are you with your work environment?	
How satisfied are you with the subject of your work?	
How satisfied are you with the level of education provided to you?	
How satisfied are you with the ability to control your work-related decisions?	
How satisfied are you with the ability to participate in the decision-making process in your work?	
**Factors related to salary**	Very dissatisfied (1)-Very satisfied (5)
How satisfied are you with your salary/remuneration level and any additional earnings/benefits?	
How satisfied are you with your pension and insurance coverage?	
How satisfied are you with your work environment?	
How satisfied are you with your absenteeism policies?	
**Factors related to relationships with colleagues**	Very dissatisfied (1)-Very satisfied (5)
How satisfied are you with the existence of strong interpersonal relationships in your work?	
How satisfied are you with the spirit of teamwork in your place of work?	
How satisfied are you with the stimulation of pride and respect as a result of work?	
How satisfied are you with the assessment of the employee's role?	
How satisfied are you with the support from the supervisory staff?	
How satisfied are you with the support from your supervisor when problems arise?	
How satisfied are you with the equal treatment of employees?	
How satisfied are you with the opportunities given for creative expression?	
**Factors related to work achievements**	Very dissatisfied (1)-Very satisfied (5)
How satisfied are you with the importance of your work?	
How satisfied are you with the level of respect for employees in your workplace?	
How satisfied are you with the recognition of your efforts/achievements?	
How satisfied are you with your achievement?	
How satisfied are you with your growth and development potential?	
How satisfied are you with the way you handle your work achievements?	

## Appendix

Please mark the number to indicate the extent to which you are satisfied with the following factors.
